# Safety and Effectiveness of Tofacitinib in Treating Polyarticular Course Juvenile Idiopathic Arthritis

**DOI:** 10.7759/cureus.48258

**Published:** 2023-11-04

**Authors:** Nicholas T Jones, Camryn L Keller, Raegan B Abadie, Matthew B Bratton, Emmilee J Henderson, Peyton Moore, Ross Rieger, Shahab Ahmadzadeh, Sridhar Tirumala, Giustino Varrassi, Sahar Shekoohi, Alan D Kaye

**Affiliations:** 1 School of Medicine, Louisiana State University Health Sciences Center, Shreveport, USA; 2 Anesthesiology, Louisiana State University Health Sciences Center, Shreveport, USA; 3 Pain Medicine, Paolo Procacci Foundation, Rome, ITA

**Keywords:** arthritis, efficacy, polyarticular course juvenile idiopathic arthritis, safety, tofacitinib

## Abstract

Polyarticular course juvenile idiopathic arthritis (pcJIA) is a form of arthritis that affects at least five joints at a time and presents before the age of 16. Its most common symptoms are pain, swelling, redness, and a limited range of motion, making it incredibly difficult for patients diagnosed to function in daily life. Historically, the leading treatment options have consisted of non-steroidal anti-inflammatory drugs (NSAIDs) and disease-modifying antirheumatic drugs (DMARDs) such as methotrexate. However, these drugs have serious toxic side effects associated with long-term use in addition to being ineffective in refractory cases. Recently, small molecule biologics have emerged as an alternate treatment to pcJIA. Tofacitinib is a small molecule JAK inhibitor that blocks the JAK/STAT cascade and decreases the transcription of genes responsible for immune function. We conducted a risk-benefit analysis to determine the viability of tofacitinib as a treatment for pcJIA. In our review, we found the side effect profile of tofacitinib to be relatively mild, with many of the serious adverse side effects occurring in those immunocompromised and those with impaired renal and hepatic metabolism. Overall, we have determined that tofacitinib has the potential to be effective in reducing flare-ups and lowering erythrocyte sedimentation rate (ESR) in immunocompetent patients with pcJIA. Additionally, our review has found that tofacitinib has the potential to be effective in patients who are refractory to traditional treatment. However, large-scale clinical trials are needed to determine if this effect holds true in younger pediatric populations, as limited data surrounds this demographic.

## Introduction and background

Rheumatoid arthritis (RA) is an umbrella term used to classify several autoimmune subtypes that primarily affect the joints. While RA affects adults, juvenile idiopathic arthritis (JIA) is the pediatric counterpart. JIA consists of seven subgroups, all characterized by arthritis in at least one or more joints before the patient's sixteenth birthday [1]. As a further subclassification of JIA, polyarticular course juvenile idiopathic arthritis (pcJIA) is classically defined as arthritis of at least five joints that presents before 16 years of age. This condition includes several other subtypes, such as rheumatoid factor (RF)-positive or RF-negative polyarthritis, enthesitis-related arthritis (ERA), extended oligoarthritis, and systemic without active systemic features [1-3]. The pathophysiology differs significantly between RF-positive and RF-negative polyarthritis. The subtypes listed here will be under the pcJIA umbrella for this review. The RF-positive subtype presents in late childhood with rapidly progressive symmetric small-joint polyarthritis [1]. In normal immune system physiology, the mature plasma cells produce immunoglobins (Ig), which are antibodies that aid in adaptative immunity against pathogens. There are five classes of Ig molecules (IgG, IgA, IgM, IgE, IgD), with IgG being the most abundant in the serum. RF is the abnormal production of the IgM molecule that binds to the natural IgG circulating in the serum. This production causes the self-reactive antibodies to bind to circulating antibodies, creating an immune complex that deposits into the synovium of joints, a classic example of a type III hypersensitivity reaction. RF-positive JIA accounts for less than 5% of all patients with JIA and remains pathophysiologically analogous to RF-positive RA in adults [1]. This subtype affects females more than males, at a ratio of 9:1 [4]. RF-negative polyarthritis accounts for around 20% of all JIA cases and presents at least two clinically distinct phenotypes [1]. The difference in presentation revolves around the presence or absence of antinuclear antibodies (ANA). ANA are autoimmune antibodies that attack a cell's nuclear material and are present in several inflammatory autoimmune disorders. In JIA, the ANA-positive and RF-negative pcJIA subtype is correlated with a worse prognosis and enhanced risk of complications compared to the ANA-negative RF-negative pcJIA subtype [4]. Several epidemiological studies have tried to pinpoint the prevalence of JIA; however, the numbers vary significantly between ethnic groups and geographical locations [5]. Studies have demonstrated the prevalence worldwide to be between 15 and 400 per 100,000 children, affecting females three times more than males. Of those affected by JIA, 15%-25% present with the polyarticular subtype, which will be discussed further in this review [4,5]. Currently, the treatment of choice for JIA depends on the clinical paradigm and classification of a patient's pcJIA. Many treatment choices exist across JIA subcategories, including non-steroidal anti-inflammatory drugs (NSAIDs), disease-modifying antirheumatic drugs (DMARDs), and biologic DMARDs. Biologics include medications that are small molecule inhibitors or monoclonal antibody treatments, such as tofacitinib [6-8]. It is well-known in the literature that some biological DMARDs have many side effects that increase the risk of complications [9]. Tofacitinib is a Janus-associated kinase (JAK) inhibitor approved for several rheumatic diseases [10]. This review assesses the current usage of tofacitinib in patients with pcJIA, emphasizing safety and efficacy. 

## Review

Tofacitinib overview

Mechanism of Action

JIA is a progressive autoimmune disease affecting children under the age of 16. While its mechanism of action is poorly understood, previous treatments have attempted to reduce inflammation by inhibiting TNF alpha and IL-6 [11,12]. However, these medications have been noted to be ineffective in many patients. Understanding of the inflammation pathway has expanded, and novel drugs have been created to target specific points in this pathway. The JAK pathway has only recently been understood. It consists of a family of receptor tyrosine kinases that are an integral part of the signaling of cytokines that bind to cytokine receptors type I and II [11]. When a cytokine binds to its receptor, it causes the receptor to dimerize. The dimerization of the receptor activates JAK, a signaling protein, to phosphorylate the receptor. The phosphorylated receptor enables the binding and phosphorylation of signal transducer and activator of transcription (STAT), a protein responsible for intracellular signaling [10]. The phosphorylated STAT can then dimerize, dissociating from the receptor and traveling to the nucleus. Once in the nucleus, STAT activates the transcription of genes involved in immune cell division, survival, and recruitment. Tofacitinib is a JAK inhibitor that blocks the JAK/STAT cascade and decreases the transcription of genes responsible for immune function.

Pharmacodynamic Considerations

The JAK inhibitor tofacitinib has classically been administered orally. The pharmacokinetic profile of tofacitinib includes a relatively short half-life of three hours, reaching a peak plasma concentration within one hour [13]. In one study, a dose of 5 mg twice daily was approved to be well tolerated, with the maximum plasma concentration increased in the fed state. However, its efficacy can be affected by patient compliance. As a result, a novel extended-release (ER) version of the drug was created. This tablet version contains hydroxyethyl cellulose, which restricts the amount of tofacitinib released out of a hole in the coating of one end of the tablet, and sugar, which provides the osmotic driving force for water [13]. In a randomized, cross-over study conducted by Pfizer, which examined the difference between standard-release and extended-release tablets, it was discovered that the area under the plasma concentration curve was equal for similar doses with no increase in side effects [14]. Consequently, the ER tablet may benefit patients who struggle with medication compliance.

Drug Interactions and Elimination

Tofacitinib has several known drug interactions. The drug is metabolized in the liver through an oxidation reaction followed by N-demethylation through a group of hepatic enzymes known as cytochromes (CYP), specifically CYP3A4 and CYP2C1 [15]. As a result, tofacitinib is contraindicated in patients taking medications that also use these liver enzymes, such as clopidogrel and omeprazole [16]. Thus, it is essential to consider similar interactions that may render the drug ineffective. After metabolism by the liver, the byproducts are excreted in the kidneys. In patients experiencing renal failure, the excretion rate of tofacitinib may be decreased. Renal failure is relatively common in patients with RA due to the chronic intake of nephrotoxic medications such as NSAIDs and methotrexate. A study by Bae SH et al. found a significant increase in the area under the drug metabolism curve in rats with renal failure compared to control rats [17].

Methods

The search for clinical trials investigating tofacitinib for the treatment of pcJIA encompassed three databases: Google Scholar, PubMed, and the ClinicalTrials.gov database. We ultimately selected four studies to be included in the review tables to emphasize both efficacy and side effects: two of these are completed clinical trials, and two are currently recruiting. The inclusion criteria for studies were that that they had to directly assess the effects of tofacitinib on pediatric populations with pcJIA and whether those effects were beneficial or adverse. Exclusion criteria included studies older than 10 years. Additionally, one case study was selected to showcase a refractory case of pcJIA.

Safety and long-term use

Adult Side Effects

Long-term use of tofacitinib for autoimmune inflammatory conditions predisposes adult patients to numerous adverse effects. Multiple studies have shown that while taking tofacitinib over nine years, more than 25% of patients experience serious adverse effects, with one study revealing that 23.1% of patients discontinued their treatment due to adverse effects [18,19]. The most common type of unfavorable effect for both low-dose (5 mg) and high-dose (10 mg) tofacitinib is infection, with one study finding that over 67% of patients experienced at least one infection while using the drug [18]. Some of the most common infection subtypes among these patients include upper respiratory and urinary tract infections; however, the most common serious infection events were pneumonia and herpes zoster. In most infections, high doses of tofacitinib produced higher rates of adverse effects for these infections [18,19]. Infection was also the most common adverse effect contributing to patient discontinuation of tofacitinib, with various benign and malignant neoplasms as other causes of discontinuation. Malignancies, excluding non-melanoma skin cancer (NMSC), occurred more frequently in patients receiving a high dose of tofacitinib, and the risk of developing a malignancy remained constant over five years [19,20]. The most common forms of malignancy were breast, lung, and lymphoma. These malignancies were one of the most common causes of death in addition to infection and cardiovascular events such as myocardial infarction and stroke.

Childhood Side Effects

The incidence and type of adverse effects associated with tofacitinib differ between adults and children. In one study, 37% of pcJIA patients taking tofacitinib over 24 weeks experienced mild adverse effects. Most of these adverse effects occurred within the first six weeks of initiating treatment and gradually improved throughout the 24 weeks. The most common adverse effect was vomiting, which occurred in almost 15% of patients [21]. Headache and elevated alanine transaminase were other side effects noted in the first six weeks; however, both conditions resolved over the following weeks. Anemia was an adverse effect found in multiple studies. It appeared more frequently over the first 24 weeks, increasing from 3.7% at week six to 7.4% at week 24 [21,22]. Unresolved viral infections at the end of the study were a common moderate adverse effect [22]. The adverse effects associated with long-term tofacitinib in children are less prevalent and milder than those seen in adults, and many of these adverse effects resolve over time.

Contraindications of Tofacitinib

Tofacitinib can have many contraindications, mainly for those who have immunosuppression and serious hepatic failure. Tofacitinib is not recommended for individuals with hepatic impairment because of the risk of increasing immunosuppression and increased hepatic metabolism [23]. As discussed previously, immunosuppressed patients should not receive tofacitinib because of the risk of infection. Those who have hepatic failure are at an increased risk of infection. This drug should not be used in patients with a lymphocyte count of less than 500 cells/mm3, an absolute neutrophil count of less than 1,000 cells/mm3, or those with hemoglobin levels of less than 9 g/dL. Concurrent use of medications that are CYP3A4 inducers can cause tofacitinib to have a lessened clinical response; therefore, dosing should be monitored [10,23]. Tofacitinib is contraindicated in patients with active infection. Tofacitinib is a pregnancy category C drug. In rats and rabbits, tofacitinib has teratogenic and feticidal effects [10].

Efficacy of tofacitinib for pcJIA

In multiple studies, most pcJIA patients receiving tofacitinib showed improvement in the clinical symptoms of arthritis. Some patients showed reduced symptoms as early as two weeks after starting treatment [24]. One study utilized the number of flare-ups as a measurement to evaluate the efficacy of tofacitinib. A flare-up is defined by the Pediatric Rheumatology Collaborative Study Group (PRCSG) as at least three of the six core variables worsening by 30% or more on the Childhood Health Assessment Questionnaire (CHAQ) scale. These core variables include physician assessment, parent/patient assessment, functional ability, number of joints active in disease, number of joints with limited range of motion, and erythrocyte sedimentation rate (ESR) [12,24]. In a study by Ruperto N et al., patients using tofacitinib showed significantly reduced flare-ups. The flare-up rate of patients taking tofacitinib was 29%, whereas the flare-up rate in patients in the placebo group was significantly higher at 75%. Additionally, patients using tofacitinib experienced a more extended time between flare-ups when compared to a placebo group. However, clinical remission, defined as six months of continuous inactive disease, occurred in less than 5% of patients [24]. One study used the juvenile arthritis disease activity score (JADAS-10) to show that over 97% of patients showed improved physical function and decreased the number of joints involved after introducing tofacitinib [25]. In addition, they found that over 24 weeks, the dosages of corticosteroids and other anti-inflammatory drugs could be reduced and, in some cases, discontinued. The ESR and platelet count were significantly decreased compared to control groups, lowering inflammation in pcJIA patients. Additionally, the safety of placebo and tofacitinib groups were similar [25]. These studies concluded that tofacitinib significantly improved the clinical symptoms of pcJIA and introduced no new safety risks to patients, reflecting a favorable risk-benefit analysis. One case study highlighted a patient with pcJIA who was resistant to treatment, affecting multiple joints, and being treated with a combination of a corticosteroid, NSAID, and DMARD. However, symptoms improved over two months when this patient added tofacitinib to the treatment plan. Additionally, after six months, the patient could discontinue all other medications except tofacitinib. The patient's ESR decreased to a level within the normal range, and their JADAS-10 score lowered, reflecting a mildly active disease state [25]. These results show that tofacitinib can decrease symptom severity in patients with pcJIA, which is refractory to other treatment options.

Trials for JIA and pcJIA

Since the development of DMARDs, such as tofacitinib, there have been multiple clinical trials trying to display the efficacy and side effects of tofacitinib in various diseases. Table 1 shows two completed clinical trial studies on the use of tofacitinib in patients with JIA. Ruperto N et al. (2017) focused on the pharmacokinetics of the medication in the body and its side effects, while Ruperto N et al. (2021) focused on the clinical efficacy of reducing flare-ups in patients with JIA. These are the only two completed studies focusing on JIA and tofacitinib in pediatric populations found on the clinicaltrials.gov database. Table 2 presents ongoing clinical trials involving tofacitinib for JIA as listed in the ClinicalTrials.gov database. The first study, detailed in Table 2, aims to enhance the current literature on the long-term use of tofacitinib in JIA, with a particular focus on its adverse effects. The second study in Table 2 seeks to broaden the application of tofacitinib to other conditions that fall under the JIA umbrella, such as systemic JIA.

**Table 1:** Completed clinical trials of tofacitinib in pcJIA populations. pcJIA: Polyarticular course juvenile idiopathic arthritis; JIA: Juvenile idiopathic arthritis; NSAIDs: Non-steroidal anti-inflammatory drugs.

Author, Year	Study Population	Results and Findings	Conclusions
Ruperto N et al., 2017 [22]	Patients aged 2 to 18 years and currently diagnosed with JIA. If treated, the patients must be on stable doses of NSAIDs, corticosteroids, or methotrexate.	Clearance and volume of distribution of tofacitinib was shown to decrease with age and have no adverse side effects that resulted in discontinuation of the medication.	In conclusion, the study confirms dosing regimens and acceptable taste for further studies to investigate efficacy.
Ruperto N et. al., 2021 [24]	Patients aged 2 to 18 years old and currently diagnosed with JIA and patients being treated should be on stable methotrexate.	The study found that flare-up rates are significantly lower in the tofacitinib group (29%) compared to placebo (59%). Adverse events reported in 77% of the tofacitinib group, while only 74% of placebo group.	This study concluded that tofacitinib is an effective treatment for JIA in pediatric populations.

**Table 2:** Recruiting clinical trials of tofacitinib in JIA patients. JIA: Juvenile idiopathic arthritis.

Study Title	Proposed Study Population	Outcomes
A long-term, open-label follow-up study of tofacitinib for treatment for juvenile idiopathic arthritis (JIA)	Pediatric patients with JIA, aged 2 to under 18 years, who have completed a previous study on tofacitinib in patients with JIA.	Adverse events, safety, body weight, height and tanner stages will all be recorded for the duration of the study, up to 8 years. In addition, there are various secondary measurements outlined.
Efficacy, safety, tolerability and pharmacokinetics of tofacitinib for the treatments of systemic juvenile idiopathic arthritis (sJIA) with active systemic features in children and adolescent subjects	Pediatric patients from 2 to 17 years who have not been previously treated with tofacitinib for JIA and are on a stable dose of either methotrexate or oral prednisone.	The study plans to measure the time to flare-up for up to 82 weeks after randomization.

## Conclusions

pcJIA affects a significant portion of the population under 16, with a higher ratio of females to males. Although no cure exists, using tofacitinib for treating autoimmune diseases such as RA and pcJIA is common practice to reduce a patient's symptoms, especially in refractory cases. This review expresses the sentiment that tofacitinib can be used for effective symptomatic treatment, reducing flare-ups and ESR. Tofacitinib has been used for JIA refractory to other therapies, including DMARDs. Additionally, the safety profile of tofacitinib is relatively mild, with severe adverse effects occurring in a minority of cases. Overall, there are contraindications for using the medication in patients with impaired hepatic metabolism, impaired renal elimination, and immunosuppressed patients. This review has highlighted the potential effectiveness and safety of tofacitinib over long-term use. However, this review is limited by the relatively insufficient data that establish the safety and efficacy of tofacitinib in pediatric populations with JIA. This medication may be used in adult and pediatric populations with a low risk of serious adverse side effects and increased effectiveness when other DMARDs have failed.
